# Author Correction: Deep-learning two-photon fiberscopy for video-rate brain imaging in freely-behaving mice

**DOI:** 10.1038/s41467-022-33164-5

**Published:** 2022-10-03

**Authors:** Honghua Guan, Dawei Li, Hyeon-cheol Park, Ang Li, Yuanlei Yue, Yung-Tian A. Gau, Ming-Jun Li, Dwight E. Bergles, Hui Lu, Xingde Li

**Affiliations:** 1 Department of Electrical and Computer Engineering, Johns Hopkins University, Baltimore, MD 21218 USA; 2 Department of Biomedical Engineering, Johns Hopkins University School of Medicine, Baltimore, MD 21205 USA; 3 Department of Pharmacology and Physiology, School of Medicine and Health Sciences, George Washington University, Washington, DC 20052 USA; 4 Solomon H. Snyder Department of Neuroscience, Johns Hopkins University School of Medicine, Baltimore, MD 21205 USA; 5 Science and Technology Division, Corning Incorporated, Corning, NY 14831 USA; 6 Johns Hopkins Kavli Neuroscience Discovery Institute, Baltimore, MD 21218 USA

**Keywords:** Neuroscience, Optical techniques, Multiphoton microscopy

Correction to: *Nature Communications* 10.1038/s41467-022-29236-1, published online 22 March 2022

In this article the author name Yung-Tian A. Gau was incorrectly written as Yungtian A. Gau. The original article has been corrected.

In this article the funding from Bisciotti Foundation through Johns Hopkins Technology Ventures was omitted. The original article has been corrected.

