# Supplementary material for: Deep-learning two-photon fiberscopy for video-rate brain imaging in freely-behaving mice
Source: Nat Commun. 2022 Mar 22;13:1534. doi: 10.1038/s41467-022-29236-1 (PMC8940941; doi:10.1038/s41467-022-29236-1)
Supplement: Supplementary file 1 — Supplementary Information [file 41467_2022_29236_MOESM1_ESM.pdf]

Supplementary Information for

Deep-learning two-photon fiberscopy for video-rate brain imaging in freely-behaving mice

Honghua Guan[1,*], Dawei Li[2,*], Hyeoncheol Park[2], Ang Li[2], Yuanlei Yue[3], Yungtian A Gau[4], Ming-Jun Li[5], Dwight E Bergles[4,6], Hui Lu[3] & Xingde Li[1,2,6]

[1]Department of Electrical and Computer Engineering, Johns Hopkins University, MD, 21218, USA

[2]Department of Biomedical Engineering, Johns Hopkins University School of Medicine, MD, 21205, USA

[3]Department of Pharmacology and Physiology, School of Medicine and Health Sciences, George Washington University, DC, 20052, USA [4]Solomon H. Snyder Department of Neuroscience, Johns Hopkins University School of Medicine, MD, 21205, USA

[5]Science and Technology Division, Corning Incorporated, NY, 14831, Corning, USA

[6]Johns Hopkins Kavli Neuroscience Discovery Institute

[*]Corresponding author. E-mail: xingde@jhu.edu

This PDF includes:

Supplementary Figure 1-10

Supplementary Table 1-2

SUPPLEMNTARY MATERIALS

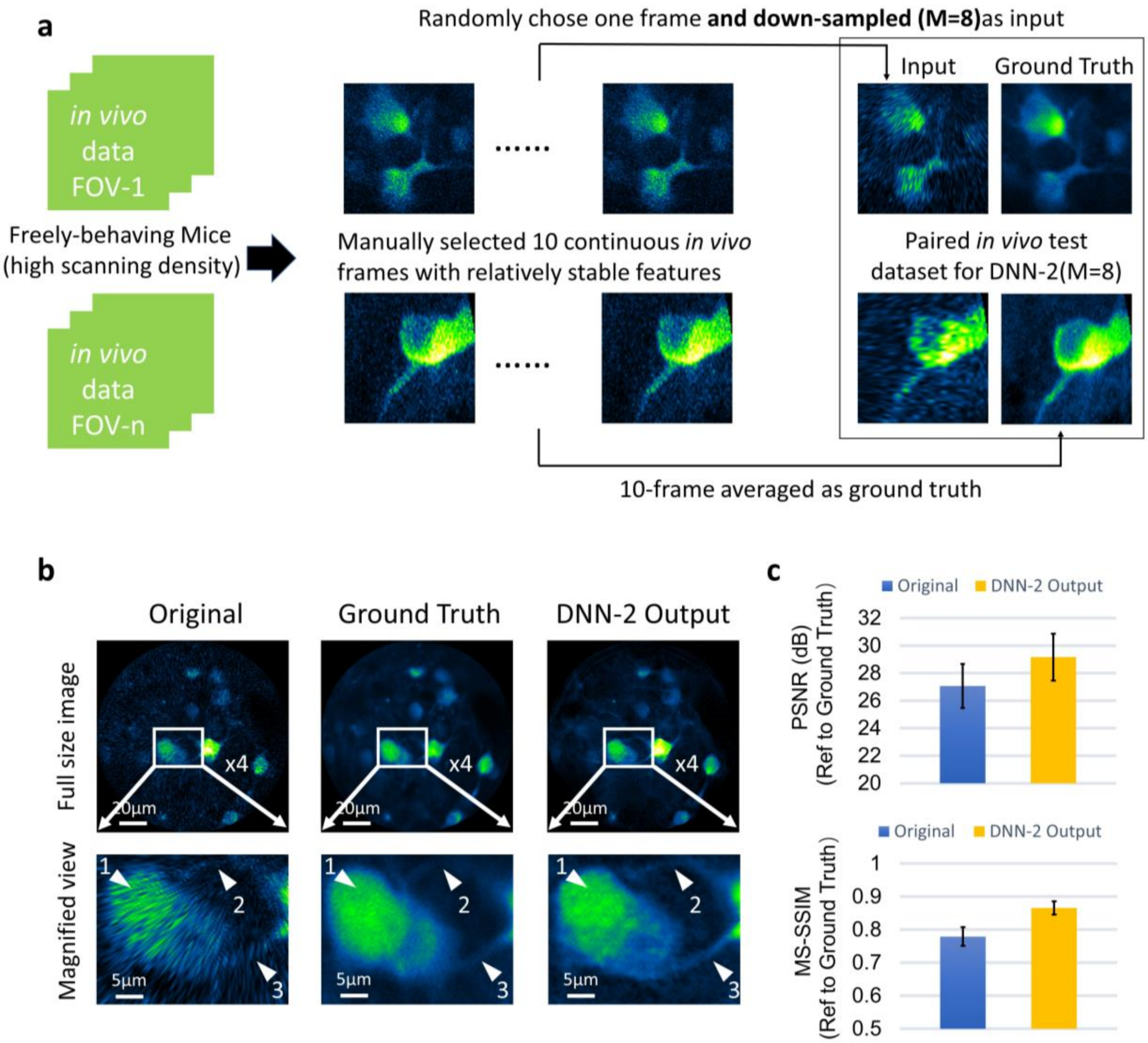

Supplementary Figure 1 | Authenticity test of DNN-2 when applied to *in vivo* images from freely-behaving mice. (a) The process for generating the authenticity test dataset for DNN-2. We acquired *in vivo* image over various FOVs in different **freely-behaving** mice at a high scanning density (corresponding to 512 spirals/frame) and manually selected 10 continuous frames that exhibited no obvious dynamic changes during the 10-frame period of time. These 10 frames were then averaged to serve as the approximate *in vivo* ground truth, and one of the 10 frames was down-sampled by a factor M=8 as an example (corresponding to a scanning density of 64 spirals/frame) to serve as the input for testing the DNN-2 that was trained with synthesized ground truth provided by DNN-1. Unlike *ex vivo* datasets, frame averaging is applicable to only a limited number of *in vivo* images. Therefore, this procedure is not practical for generating a sufficiently large training dataset and the resulted frame-averaged images can only serve as the approximate ground truth. (b) Comparison of the DNN-2 output

image with the corresponding ground truth for a representative *in vivo* input image along with a magnified region. The results demonstrated that the trained DNN-2 enabled to restore structural details which were consistent with the ground truth. The profile of a neuron somas (arrowhead 1) and dendrites (arrowhead 2 and 3) could be clearly recognized in the DNN-2 output images (and confirmed by the ground truth), but they were difficult to resolve in the original (testing input) images. (c) Global image quality comparison between the original (testing input) image and the DNN-2 output image in terms of peak signal-to-noise ratio (PSNR) and multi-scale structural similarity index measure (MS-SSIM). Data are presented as mean values (color columns) ± standard deviations (error bars). As shown in (c), DNN-2 improved the PSNR by about 2.08 dB (from 27.08 to 29.16 dB) and the MS-SSIM from 0.78 to 0.87 (with respect to the ground truth). The measurements were made over the testing dataset (with the number of samples n=10).

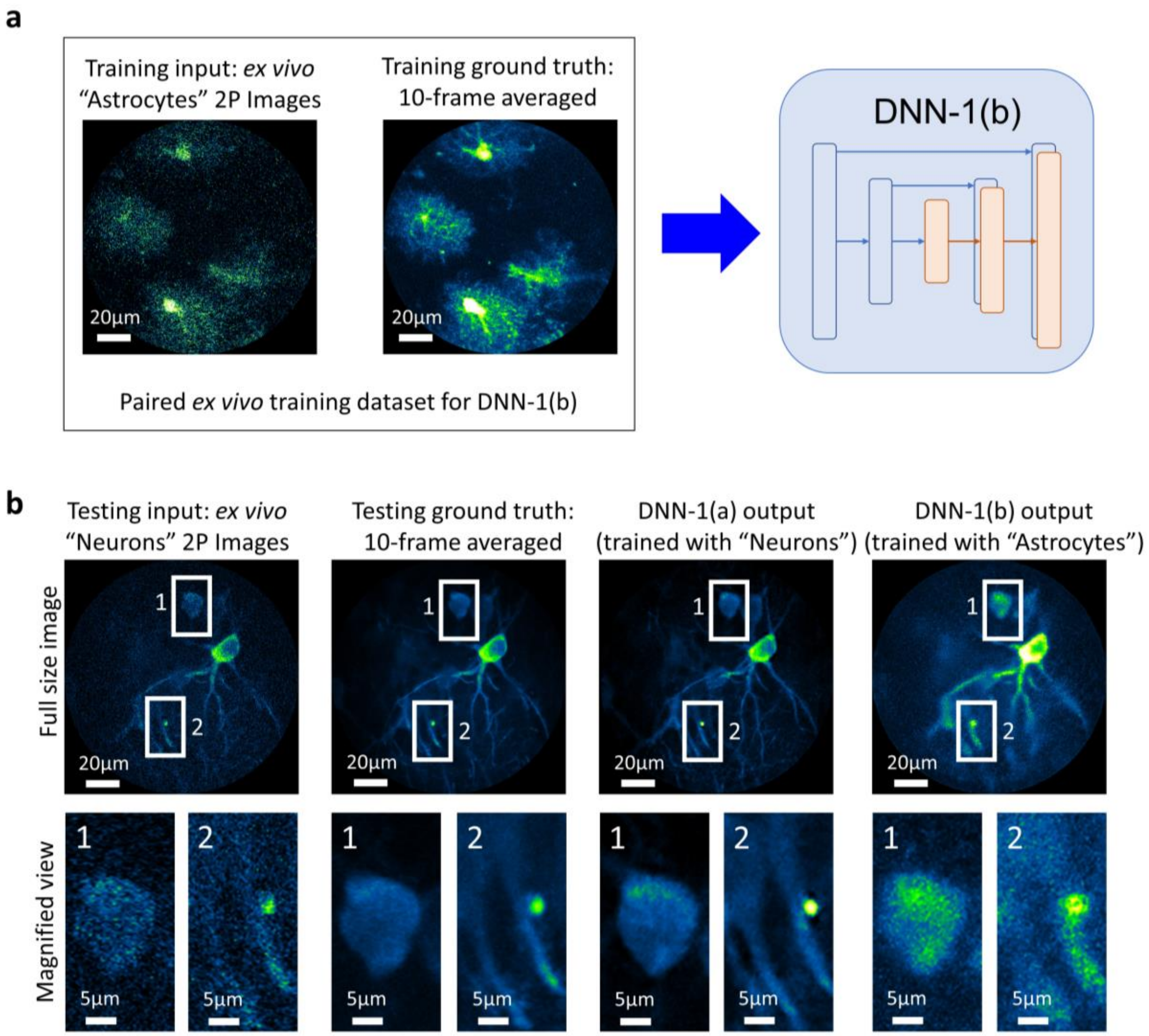

Supplementary Figure 2 | Training DNN-1with images of different cell types. (a) Training DNN-1 with 2P fiberscopy images acquired from *ex vivo* mouse brain slices of GCaMP6s-expressing astrocytes and the as-trained neural network is denoted as DNN-1(b). The data acquisition and training protocol was the same as the one described in the manuscript for training the DNN-1 with images acquired from *ex vivo* mouse brain slices of GFP-immunostained neurons and the resulted trained network is denoted as DNN-1(a) here. (b) Representative test results when applying the trained DNN-1(a) and DNN-1(b) to GFP-immunostained neuron images collected from *ex vivo* mouse brain slices. It is noted that the testing data were excluded from the training set for DNN-1(a) and DNN-1(b). Comparing with the testing input, the DNN-1(b) output shows less noise (as shown in the full-size image); but fine features in the DNN-1(b) output image are distorted when compared with the ground truth (and DNN-1(a) output) as shown in the magnified views, e.g., the soma edge is blurred (indicated by ROI 1), and the dendrite and dendritic spine structures (indicated by ROI 2) are difficult to resolve.

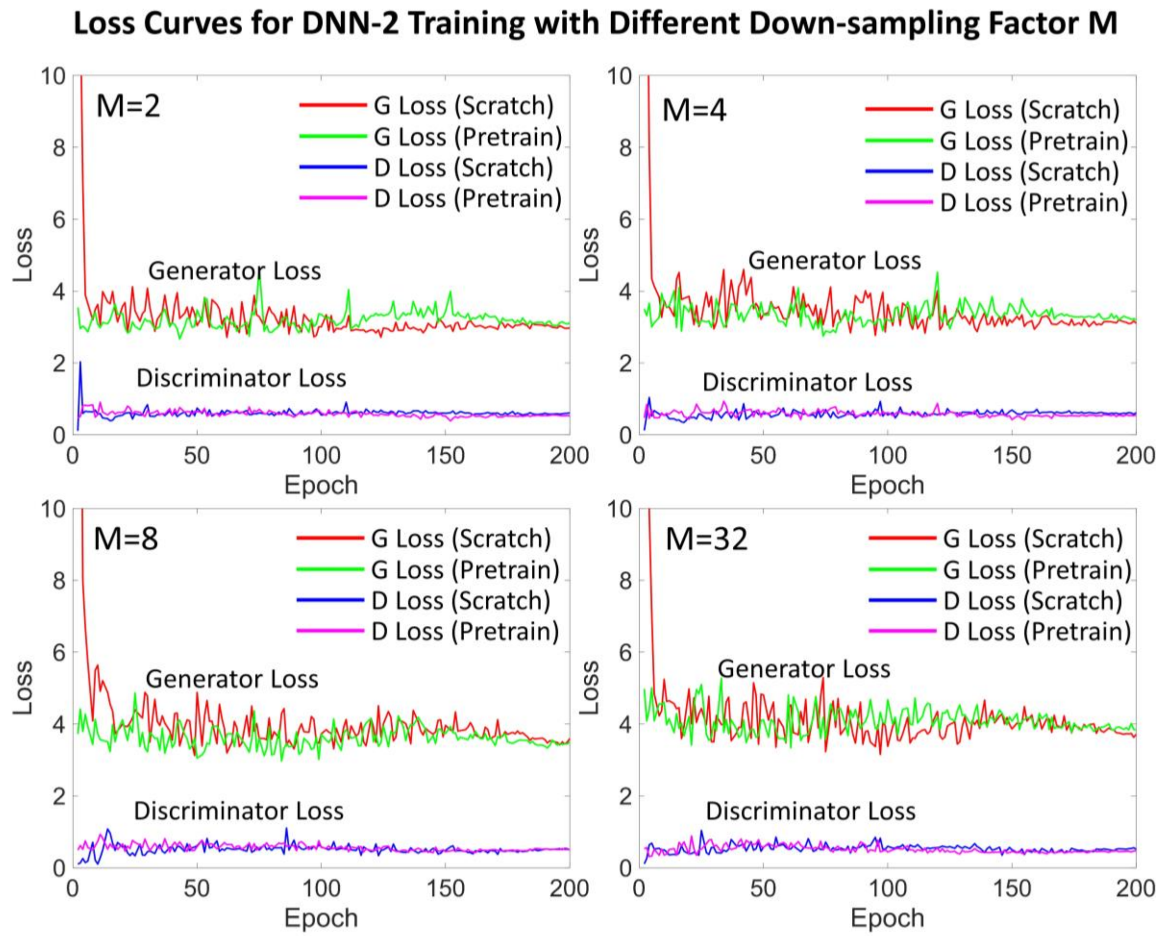

Supplementary Figure 3 | Comparisons of DNN-2 training performance without/with weight transfer from DNN-1. Loss curves of the generator and the discriminator (denoted as "G loss" and "L loss", respectively) correspond to two training configurations: 1) Training DNN-2 from scratch (i.e., initializing the generator and discriminator of DNN-2 with random weights, denoted as "Scratch"); 2) Training DNN-2 using weights from a pre-trained model (i.e., initializing the generator and discriminator of DNN-2 with weights inherited from the trained DNN-1, denoted as "Pretrain"). We chose the down-sampling factor M=2, 4, 8, and 32 as examples. As shown in the plots, the "Pretrain" method has faster convergence at the beginning (0-40 epochs). Afterwards, the performances of the two training configurations are similar. The loss curves turn to be nearly identical at the end (after about 160-200 epochs).

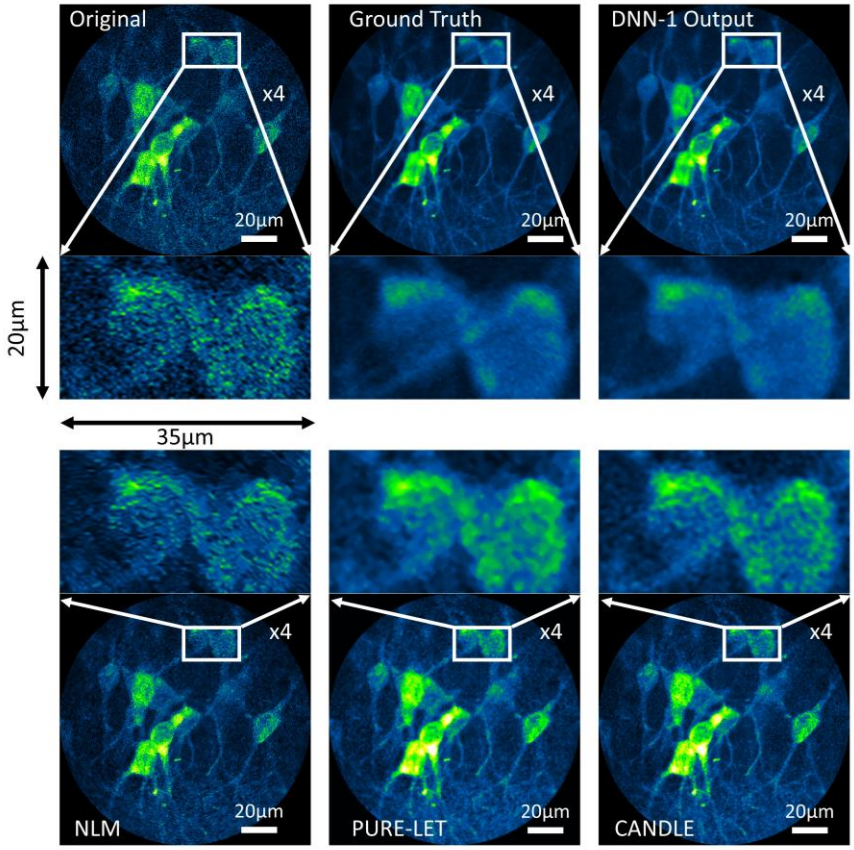

Supplementary Figure 4 | Comparison of denoising performance between DNN-1 (deep learning method) and traditional image processing methods. The testing dataset was acquired from *ex vivo* GFP-immunostained brain slices (which were excluded from the training dataset for DNN-1). The DNN-1 output shows better image quality enhancement, especially for fine features. As shown in the magnified views, the DNN-1 output remains consistent with the ground truth where the soma profile is much clearer than those obtained by the traditional image processing methods. Comparison was repeated over the whole testing dataset (with a sample size of n=80) and the results were similar. The quantitative performance comparison is given in **Table S1**.

Method	PSNR (dB)	MS-SSIM
Original	25.43	0.82
NLM[1]	28.23	0.88
PURE-LET[2]	26.38	0.89
CANDLE[3]	28.43	0.90
**DNN-1**	**30.99**	**0.92**

Supplementary Table 1 | Denoising performance among different image processing methods. The denoising performance was evaluated in terms of PSNR and MS-SSIM with respect to the ground truth (which were defined as the 10-frame averaged images).

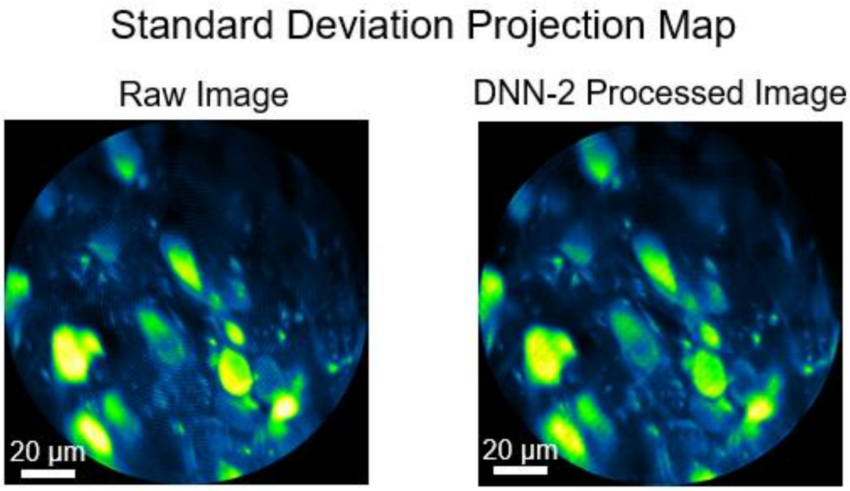

Supplementary Figure 5 | Comparison of standard deviation projection maps of the raw images and DNN-2 processed images. The raw data was acquired from freely-behaving mice at an imaging frame rate of ~26fps. The raw images were then processed by the trained DNN-2. The value for each pixel in the projection map represents the standard deviation of the time series data at that given pixel. The two maps show high structure similarity (MS-SSIM: 0.93), which implies that the proposed DL-based method maintains a high fidelity of the raw images (and the neuron firing activities).

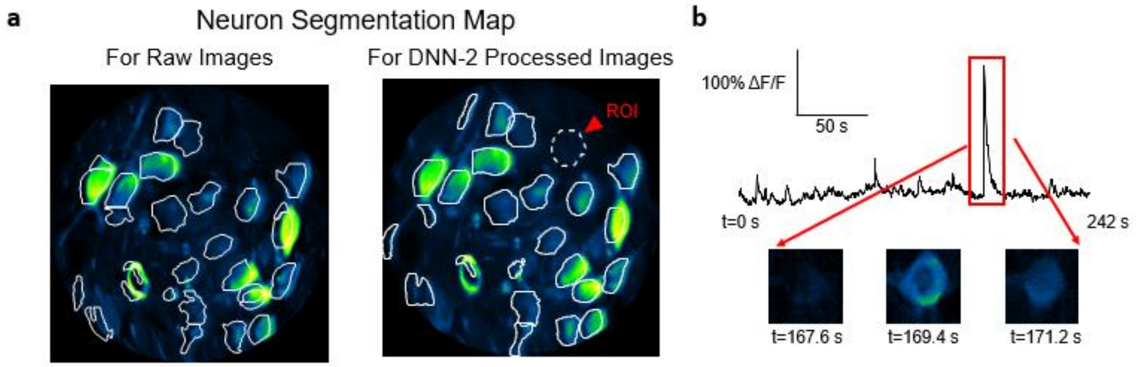

Supplementary Figure 6 | Comparison of neuron segmentation maps obtained from raw images and DNN-2 processed images. (a) The two segmentation maps correspond to the neuron segmentation results from the raw images (DNN-2 input) and the DNN-2 processed images, respectively. The standard deviation projection maps serve as background. We chose CaImAn as the processing pipeline for both datasets. Considering the different SNRs associated with the two datasets, we set the minimum SNR threshold to zero to make sure the pipeline can recognize all possible neurons within the field of view. The results show that the pipeline missed one neuron when processing the raw data (where the missed neuron has a weak GCaMP signal and was marked with a dashed line and indicated with a red arrowhead). Conversely, the pipeline could successfully extract this neuron from the background. (b) Time-varying GCaMP fluorescence intensity ($\Delta F/F$) of the selected neuron marked in (a). The fluorescence (calcium) signal indicates that the neuron truly exists. The representative dynamic images of the selected neuron at different time points also proved the authenticity of the firing activity.

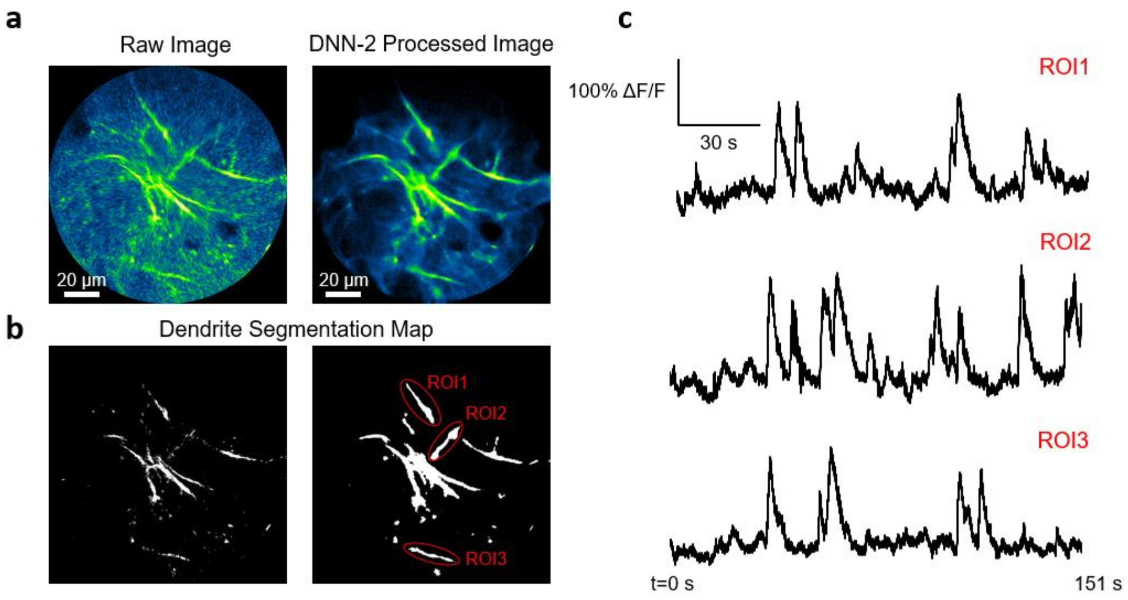

Supplementary Figure 7 | Comparison of dendrite segmentation by using raw image and DNN-2 processed image. (a) A representative *in vivo* raw image (DNN-2 input) of dendrites along with the DNN-2 processed image. The raw data was acquired from a freely-behaving mouse with an imaging frame rate of ~26 fps. (b) The two maps correspond to the segmented dendritic structures from the raw image and the DNN-2 processed image, respectively. Here we applied the maximum entropy threshold approach[4] for both images. The segmentation map from the raw image exhibits higher noise and some discontinuities in the dendritic structures, while the segmentation map from the DNN-2 processed image shows much higher image quality with much clearer dendritic profiles. (c) Time-varying GCaMP fluorescence intensity ($\Delta F/F$) curves corresponding to several representative ROIs marked in (b). The fluorescence (calcium) dynamic signals suggest that the extracted dendrite features truly exist.

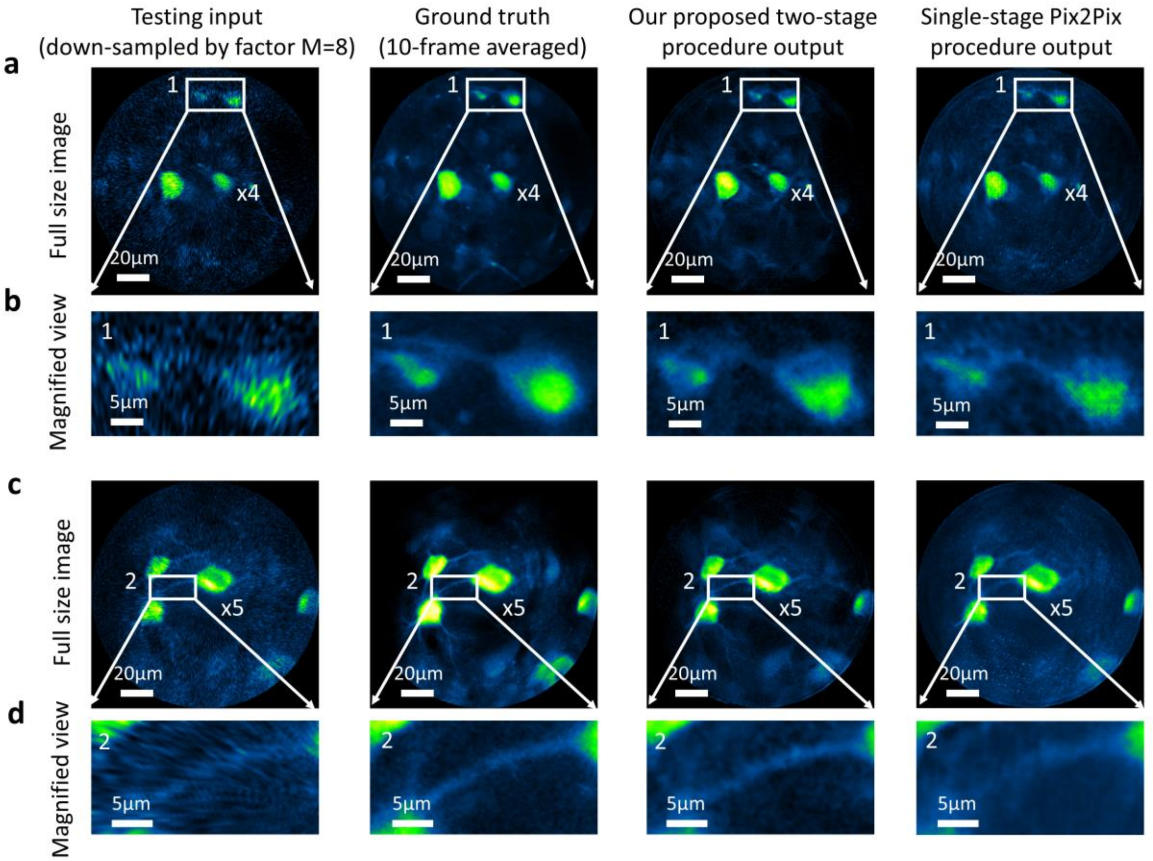

Supplementary Figure 8 | Comparison of output image quality between our proposed two-stage (DNN-1 and DNN-2) training procedure and a single-stage Pix2Pix training procedure. For the single-stage Pix2Pix training[5], we used the same ground truth as for DNN-1 training (i.e., 10-frame averaged *ex vivo* 2P fiberscopy images from mouse brain slices of GFP-immunostained neurons over multiple FOVs), and the down-sampled (by a factor M=8) *ex vivo* images as the training input. We applied the trained Pix2Pix network and our proposed DNN-2 (M=8) to the *in vivo* testing dataset collected from freely-behaving mice. The details on how the *in vivo* testing dataset were selected can be referred to **Supplementary Information Figure S1** (and noting that these *in vivo* testing images were excluded from any network training). (a) and (c) Full-size testing input, ground truth, and trained network output images over two representative FOVs. (b) and (d) Magnified views of ROIs marked in (a) and (c), respectively. Visually, our proposed two-stage training method shows better image quality improvement, where the output images remain consistent with the ground truth and show clearer neuron soma (indicated by ROI 1) as well as dendrite (indicated by ROI 2). The output image quality was evaluated and compared in terms of PSNR and MS-SSIM with respect to the ground truth. Comparing with our proposed two-stage training procedure, the single-stage training procedure reduced the PSNR by about 2.02 dB (from 29.16 to 27.13 dB) and the MS-SSIM from 0.87 to 0.84 (with respect to the ground truth).

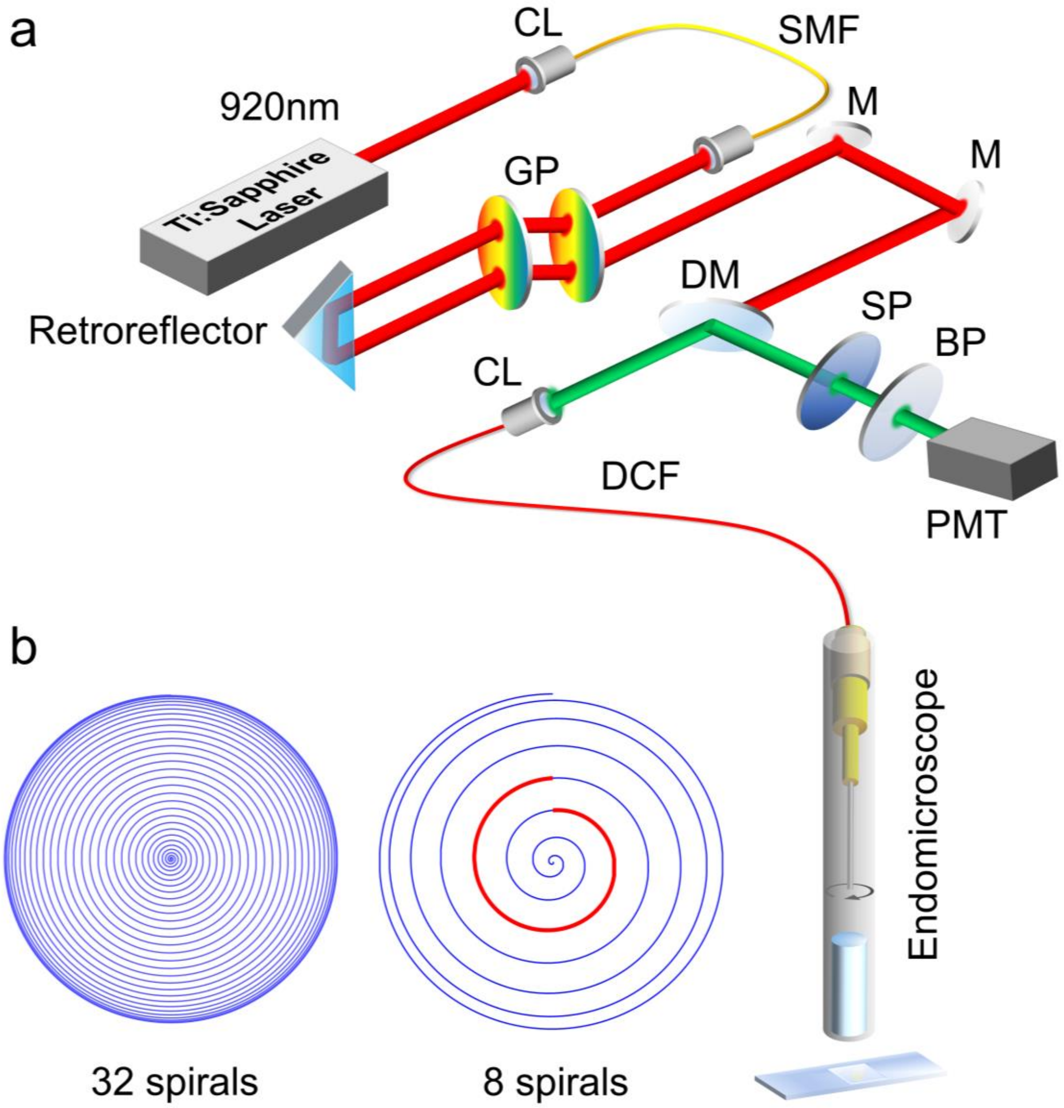

Supplementary Figure 9 | Schematic of the 2P fiberscopy imaging system. (a) System configuration: CL: coupling lens, GP: grating pairs, SMF: single-mode fiber, DM: dichroic mirror, SP: short-pass optical filter, BP: band-pass optical filter, DCF: double-clad fiber, PMT: photomultiplier. (b) Illustration of spiral scanning patterns with different spiral densities. Red curve: a representative scanning trajectory for one spiral.

Training Cycle	Initialization Method	Gain
1	Normal	0.01
2	Normal	0.02
3	Normal	0.04
4	Orthogonal	0.02
5	Xavier	0.02

Supplementary Table 2 | Initialization parameters for testing DNN-1 and DNN-2 training stability.

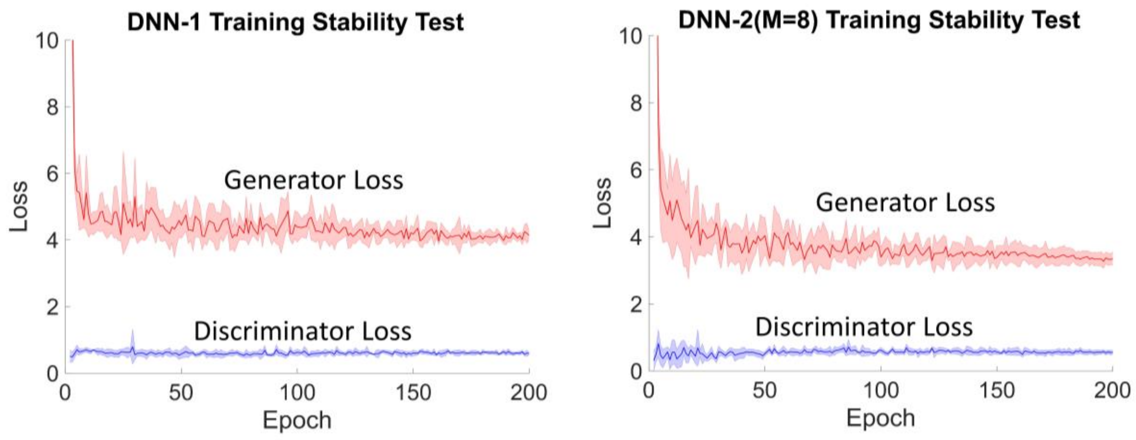

Supplementary Figure 10 | DNN-1 and representative DNN-2 (M=8) training stability test. Loss curves of the generator and the discriminator ("G loss" and "D loss") over different training cycles. The initialization parameters are listed above in **Table S2**. The red and blue solid lines represent the mean value of the G loss and D loss over different training cycles, respectively. The red and blue shaded regions correspond to the standard deviations of the G loss and D loss. The results show that the training is stable against different initialization configurations. After 150 epochs, the loss curves converged to a stable value for different initialization parameters. For DNN-1, the relative standard deviations (RSDs) of the G loss and D loss at the endpoint were 3.4% and 9.3%, respectively. For DNN-2, the RSDs of the G loss and D loss at the endpoint were 6.0% and 10.7%, respectively. For all the training in the manuscript, we chose the initialization method as "normal" with a gain of 0.02.

References

1. A. Buades, B. Coll, and J.-M. Morel, "A non-local algorithm for image denoising," in *2005 IEEE Computer Society Conference on Computer Vision and Pattern Recognition (CVPR'05)*, (IEEE, 2005), 60-65.
2. J. Li, F. Luisier, and T. Blu, "PURE-LET image deconvolution," IEEE Transactions on Image Processing **27**, 92-105 (2017).
3. P. Coupé, M. Munz, J. V. Manjón, E. S. Ruthazer, and D. L. Collins, "A CANDLE for a deeper in vivo insight," Medical image analysis **16**, 849-864 (2012).
4. L. M. Martyushev and E. Axelrod, "From dendrites and S-shaped growth curves to the maximum entropy production principle," Journal of Experimental and Theoretical Physics Letters **78**, 476-479 (2003).
5. P. Isola, J.-Y. Zhu, T. Zhou, and A. A. Efros, "Image-to-image translation with conditional adversarial networks," in *Proceedings of the IEEE conference on computer vision and pattern recognition*, 2017), 1125-1134.
