# Supplementary material for: Deep-learning two-photon fiberscopy for video-rate brain imaging in freely-behaving mice
Source: Nat Commun. 2022 Mar 22;13:1534. doi: 10.1038/s41467-022-29236-1 (PMC8940941; doi:10.1038/s41467-022-29236-1)
Supplement: Supplementary file 2 — Reporting Summary [file 41467_2022_29236_MOESM2_ESM.pdf]

nature research

Corresponding author(s): Xingde Li

Last updated by author(s): Feb 27, 2022

Reporting Summary

Nature Research wishes to improve the reproducibility of the work that we publish. This form provides structure for consistency and transparency in reporting. For further information on Nature Research policies, see our Editorial Policies and the Editorial Policy Checklist.

Statistics

For all statistical analyses, confirm that the following items are present in the figure legend, table legend, main text, or Methods section.

n/a	Confirmed	
☐	☒	The exact sample size (n) for each experimental group/condition, given as a discrete number and unit of measurement
☐	☒	A statement on whether measurements were taken from distinct samples or whether the same sample was measured repeatedly
☒	☐	The statistical test(s) used AND whether they are one- or two-sided<br>*Only common tests should be described solely by name; describe more complex techniques in the Methods section.*
☒	☐	A description of all covariates tested
☒	☐	A description of any assumptions or corrections, such as tests of normality and adjustment for multiple comparisons
☐	☒	A full description of the statistical parameters including central tendency (e.g. means) or other basic estimates (e.g. regression coefficient) AND variation (e.g. standard deviation) or associated estimates of uncertainty (e.g. confidence intervals)
☒	☐	For null hypothesis testing, the test statistic (e.g. F, t, r) with confidence intervals, effect sizes, degrees of freedom and P value noted<br>*Give P values as exact values whenever suitable.*
☒	☐	For Bayesian analysis, information on the choice of priors and Markov chain Monte Carlo settings
☒	☐	For hierarchical and complex designs, identification of the appropriate level for tests and full reporting of outcomes
☒	☐	Estimates of effect sizes (e.g. Cohen's d, Pearson's r), indicating how they were calculated

Our web collection on statistics for biologists contains articles on many of the points above.

Software and code

Policy information about availability of computer code

Data collection	<Two-photon Imaging><br>The two-photon fiberscopy data collection software was home-developed specifically for our imaging system (not available to the public).<br><br><Video Recording><br>The software for collecting the video footage of the freely-moving mice was provided by the camera manufacturer (available to any buyers).
Data analysis	The deep-learning based platform was adapted from a publicly available repository: https://github.com/junyanz/pytorch-CycleGAN-and-pix2pix. Our customized source code is available upon request.<br><br>Motion correction source codes are publicly available below:<br>NoRMCorre: https://github.com/flatironinstitute/NoRMCorre<br><br>Calcium signal analysis source codes are publicly available below:<br>CalmAn-MATLAB: https://github.com/flatironinstitute/CalmAn-MATLAB/

For manuscripts utilizing custom algorithms or software that are central to the research but not yet described in published literature, software must be made available to editors and reviewers. We strongly encourage code deposition in a community repository (e.g. GitHub). See the Nature Research guidelines for submitting code & software for further information.

Data

Policy information about availability of data

All manuscripts must include a data availability statement. This statement should provide the following information, where applicable:

- Accession codes, unique identifiers, or web links for publicly available datasets
- A list of figures that have associated raw data
- A description of any restrictions on data availability

Two-photon fiberscopy imaging datasets (including the source data and all relevant raw data) have been uploaded to the publicly accessible repository Figshare at (https://doi.org/10.6084/m9.figshare.19193792).

Field-specific reporting

Please select the one below that is the best fit for your research. If you are not sure, read the appropriate sections before making your selection.

[x] Life sciences [ ] Behavioural & social sciences [ ] Ecological, evolutionary & environmental sciences

For a reference copy of the document with all sections, see nature.com/documents/nr-reporting-summary-flat.pdf

Life sciences study design

All studies must disclose on these points even when the disclosure is negative.

Sample size	[ex vivo]<br>We used two brain slices (from two mice) for the ex vivo imaging study. Each sample had a large area and provided multiple FOVs for fiberscopy imaging, allowing us to collect a sufficient number (~100) of independent images.<br><br>[in vivo]<br>We used five GCaMP6s-expressing mice in the present study. Head-fixed images were collected from three mice over different FOVs. Freely-behaving images were collected from 2 different mice over different FOVs.
Data exclusions	[ex vivo]<br>We excluded about 20% of the two-photon fiberscopy images collected from the stained mice brain slices that did not show clear neuron structures and thus could not be used as ground truth for neural network training or testing.<br><br>[in vivo]<br>About 30% two-photon fiberscopy images collected from head-fixed mice were excluded for training the deep neural network (DNN). Those images did not show obvious neural activities or changes in neural activities. Exclusion of those images in training the DNN saved computational time.<br><br>No two-photon fiberscopy images collected from freely-behaving mice were excluded.
Replication	Our method (for SNR and spatial imaging resolution enhancement) was tested against multiple independent images collected from two freely moving mice over different FOVs.
Randomization	n/a
Blinding	n/a

Reporting for specific materials, systems and methods

We require information from authors about some types of materials, experimental systems and methods used in many studies. Here, indicate whether each material, system or method listed is relevant to your study. If you are not sure if a list item applies to your research, read the appropriate section before selecting a response.

Materials & experimental systems

n/a	Involved in the study	
[ ]	[x]	Antibodies
[x]	[ ]	Eukaryotic cell lines
[x]	[ ]	Palaeontology and archaeology
[ ]	[x]	Animals and other organisms
[x]	[ ]	Human research participants
[x]	[ ]	Clinical data
[x]	[ ]	Dual use research of concern

Methods

n/a	Involved in the study	
[x]	[ ]	ChIP-seq
[x]	[ ]	Flow cytometry
[x]	[ ]	MRI-based neuroimaging

Antibodies

Antibodies used	Chicken polyclonal anti-GFP (AVESLABS, RRID: AB_2307313), used at 1:4000. Alexa Fluor 488 AffiniPure Donkey Anti-Chicken IgY (IgG) (H+L) (Jackson ImmunoResearch, RRID: AB_2340375), used at 1:2000.<br>Other details such as the amount and temperatures can be found in the main text.
Validation	Chicken anti-GFP antibodies have been verified by AVESLABS via immunohistochemistry (1:500 dilution) in transgenic mice expressing the GFP gene product (AVESLABS: https://www.aveslabs.com/products/anti-green-fluorescent-protein-antibody-gfp).<br><br>Alexa Fluor 488 AffiniPure Donkey Anti-Chicken IgY (IgG) (H+L) antibodies have been tested by Jackson ImmunoResearch via ELISA and/or solid-phase adsorbed to ensure minimal cross-reaction with mouse serum proteins (Jackson ImmunoResearch: https://www.jacksonimmuno.com/catalog/products/703-545-155).<br><br>Both antibodies were also validated in our previous work (Agarwal et al., 2017), where it was shown to specifically label GFP-expressing cells in transgenic mice carrying GFP product.<br>The paper can be found at:<br>https://www.ncbi.nlm.nih.gov/pmc/articles/PMC5308886

Animals and other organisms

Policy information about studies involving animals; ARRIVE guidelines recommended for reporting animal research

Laboratory animals	[mouse for ex vivo imaging]<br>We used male mice C57BL/6J-Tg(Thy1-GCaMP6f)GP5.5Dkim/J (RRID: IMSR_JAX:024276) of 20- to 24-week-old for this experiment. Mice were maintained on a 12 hr light/dark cycle, and food and water was provided ad libitum.<br><br>[mouse for in vivo imaging]<br>We used male mice carrying Camk2-cre allele on the C57/B6 for this experiment. The strain was obtained from the Jackson Laboratory (JAX#005359). Animals were given ad libitum access to standard mouse chow and water, housed 4 to 5 per cage in a room of controlled temperature (23 ± 1°C) and humidity (50 ± 10%) with a 12 hr light-dark cycle.<br><br>Other details such as cranial window preparation and brain imaging protocols can be found in the main text.
Wild animals	n/a
Field-collected samples	n/a
Ethics oversight	All the animal housing and experimentation procedures were performed under the standards of humane animal care described in the National Institutes of Health Guide for the Care and Use of Laboratory Animals with protocols approved by the Institutional Animal Care and Use Committees at the George Washington University and Johns Hopkins University.

Note that full information on the approval of the study protocol must also be provided in the manuscript.
