# Supplementary material for: Deep-learning two-photon fiberscopy for video-rate brain imaging in freely-behaving mice
Source: Nat Commun. 2022 Mar 22;13:1534. doi: 10.1038/s41467-022-29236-1 (PMC8940941; doi:10.1038/s41467-022-29236-1)
Supplement: Supplementary file 3 — Description of Additional Supplementary Files [file 41467_2022_29236_MOESM3_ESM.pdf]

Title: Supplementary Movie 1
Description: SNR enhancement by DNN-1 for *in vivo* head-fixed 2P fiberscopy imaging. Left: Raw imaging data. Right: Corresponding DNN-1 output results. The raw images were acquired at ~3.3 frames/second with the scanning speed at 3360 spirals/sec and the scanning density at 512 spirals/frame. The video playback is set to 10x speedup comparing with the raw imaging frame rate.

Title: Supplementary Movie 2
Description: Spatial imaging resolution and SNR recovery by DNN-2 for *in vivo* head-fixed 2P images acquired at various scanning densities. Top left: Raw imaging data without digital down-sampling. The raw images were acquired at ~3.3 frames/second with the scanning speed at 3360 spirals/sec and the scanning density at 512 spirals/frame. Bottom left: The corresponding DNN-1 output results which served as the reference for quantitative image quality comparison with the output from DNN-2. Top right: Down-sampled imaging data with different down-sampling (or speed boost) factors *M* which served as the input for testing DNN-2. Bottom right: The corresponding DNN-2 output results. The video playbacks for each column are set to 10x speedup, 1.25x speedup and 3.2x slow down comparing with the raw image frame rate, respectively.

Title: Supplementary Movie 3
Description: Performance of DNN-2 for video-rate 2P fiberscopy brain imaging in freely-behaving mice. Left: video footage of a freely-behaving mouse during imaging acquisition. Middle: Raw imaging data Right: Corresponding DNN-2 output results. The raw images were acquired at ~26.4 frames/second with the scanning speed at 3360 spirals/sec and the scanning density at 64 spirals/frame. The video playback is set to 10x speedup comparing with the raw image frame rate.
